# Clinical characteristics, diagnosis, and predictors of neurosyphilis patients with human immunodeficiency virus co-infection: A retrospective study at infectious diseases hospitals in two cities of China

**DOI:** 10.1097/MD.0000000000027430

**Published:** 2021-10-22

**Authors:** Jianhua Yu, JinChuan Shi, Hu Wan, Jianwei Li, Ying Shao, Jiangzhu Ye, Lili Dai, Xiwen Wang, An Liu

**Affiliations:** a Hangzhou Xixi Hospital Affiliated to Zhejiang University of Traditional Chinese Medicine, Hangzhou, China; b Center for Infectious Diseases, Beijing Youan Hospital, Capital Medical University, Beijing, China; c Peking University 302 Clinical Medical School, Beijing, China.

**Keywords:** acquired immunodeficiency syndrome, clinical characteristics, human immunodeficiency virus, neurosyphilis, risk factors

## Abstract

This study aimed to compare between the clinical and laboratory characteristics of neurosyphilis and those of syphilis in human immunodeficiency virus (HIV) positive and explore the risk factors associated with the occurrence of neurosyphilis in the HIV infected.

In-patients diagnosed with HIV and syphilis co-infection who underwent a lumbar puncture and completed cerebrospinal fluid (CSF) examination were divided into neurosyphilis group and syphilis group. The demographic characteristics, symptoms and signs, and laboratory tests of the 2 groups were comparatively analyzed. Logistic regression analysis was used to explore the risk factors associated with the occurrence of neurosyphilis.

Among 81 patients, 33 patients were assigned to the neurosyphilis group, and 48 to the syphilis group. There were no significant differences in the age, gender, marital status, acquired immunodeficiency syndrome course, opportunistic infections, serum HIV viral load, and history of syphilis treatment. The difference in HIV transmission route between the 2 groups was statistically significant (*P* = .010), and the patients from the neurosyphilis group were mainly infected via heterosexual contact. The proportion of serum toludine red unheated serum test (TRUST) titer ≥1:16 in the neurosyphilis group were 78.8%, which was significantly higher compared to the syphilis group (48.9%). The level of CSF white blood cell count, CSF protein, and CSF HIV viral load in the neurosyphilis group were significantly higher than those of the syphilis group. The proportion of patients with neurological symptoms and signs in the neurosyphilis group was significantly higher compared to the syphilis group (*P* < .001). Multivariate logistic regression analysis showed that heterosexual contact transmission route, not received antiretroviral therapy, lower CD4 cell count and higher serum TRUST titer, untreated with syphilis, and neurological symptoms and signs were risk factors associated with the occurrence of neurosyphilis.

The serum TRUST titer, CSF white blood cell count, CSF protein level, CSF HIV viral load, and the percentage of neurological symptoms and signs in the neurosyphilis group were higher. Heterosexual transmission route, not received antiretroviral therapy, and untreated with syphilis prompted the possibility of neurosyphilis occurrence.

## 1 Introduction

By the end of 2017, there were 758,600 human immunodeficiency virus (HIV)/acquired immunodeficiency syndrome (AIDS) patients living in China, and 134,500 new HIV/AIDS cases were reported in 2017, an increase of 7.09% (125,600 cases) over 2016. Syphilis is a chronic sexually transmitted disease caused by *Treponema pallidum* (*TP*) infection, which can cause multi-system damage of human body including cardiovascular system, skeletal system, and nervous system. In recent years, it was the rapid growth of patients with syphilis infection in China. According to the report of an epidemiological survey, the incidence of syphilis with average annual growth rate of 13.37% during 2000 to 2013, was second only to tuberculosis, and hepatitis B, became an important public health problem.^[1]^ It was reported nationally 358,534 cases of syphilis cases in 2010, with incidence of 26.86 per 100,000 in 2010, and increased 17.02% than in 2009, listed third among B class infectious diseases of China. Syphilis did great harm to the human body, which can invade the skin mucosa, cardiovascular, nerve, bone and so on to cause multi-system damage, and can also cause abortion, stillbirth, and congenital syphilis in pregnant women.^[2]^ Neurosyphilis caused by *TP* invasion of the central nervous system (CNS), and infringe patients with meninges and blood vessels, cause nerve tissue degeneration or vascular lesions in CNS. Its clinical manifestation was complicated, the lack of specificity, and many patients were misdiagnosed or undiagnosed. Some of the symptoms included neurological impairment manifestations, which lacked specificity.^[3]^ HIV and *TP* also affected the CNS with the same transmission route among the same vulnerable population.^[4]^ HIV/AIDS and syphilis co-infection can increase the likelihood of developing neurosyphilis.^[5]^

In recent years, due to the increase of high risk behaviors among men who have sex with men, unprotected sexual behavior in the elderly, and casual sexual behavior among the youth, the infection rate of neurosyphilis in HIV/AIDS population is also increasing year by year.^[6–8]^ A review of syphilis showed that the incidence of neurosyphilis among HIV-infected patients was 2.1% compared to 0.6% among those without HIV/AIDS.^[9]^ HIV could not only directly infect CNS, but also affected CNS through the opportunistic infection or tumor caused by immunosuppression, thus complicating the clinical manifestations and diagnosis of patients and increasing the difficulty of diagnosis of neurosyphilis. This caused delaying in diagnosis and treatment of neurosyphilis infection in HIV/AIDS population. Ghanem et al^[10]^ suggested that rapid plasma regain test (RPR) titer ≥1:128, male, not receiving antiretroviral therapy (ART) were risk factors for developing neurosyphilis in syphilis patients with HIV infection. A study in Taiwan, China, showed that CD4 T lymphocyte counts (CD4 cell counts) ≤200 cells/μL was associated with failure of neurosyphilis treatment.^[11]^

With the widespread use of antibiotics in treatment of syphilis, cardiovascular syphilis, and other serious types of syphilis have been rare. However, due to hidden incidence and atypical clinical manifestations, more and more cases of neurosyphilis reported in China, which seriously harmed public health safety. Therefore, the correct understanding of neurosyphilis is of great significance to improve healthcare workers diagnosis, treatment, and control the prevalence of the disease.

To further understand the clinical characteristics of neurosyphilis in HIV/AIDS patients, and explore the risk factors associated with neurosyphilis, we undertook this retrospective study to comparatively analyze the data of HIV/AIDS patients with neurosyphilis and syphilis co-infection at 2 hospitals of infection diseases in Beijing and Hangzhou.

## 2 Methods

### 2.1 Population and ethics statement

In this study, we retrospectively reviewed the medical records of all patients diagnosed with HIV/AIDS and syphilis co-infection, who underwent a lumbar puncture and completed cerebrospinal fluid (CSF) examination from January 1, 2013 to August 31, 2017at Beijing Youan Hospital, Capital Medical University in Beijing, and Hanzhou Xixi hospital in Hangzhou, which were designated infection diseases hospitals located in Beijing and Hangzhou, respectively.

The following demographic and clinical data were collected from medical records: age, gender, marital status, possible HIV transmission route, CD4 cell count, HIV/AIDS course (HIV/AIDS course was classified as newly diagnosed [within 2 weeks], within 6 months, longer than 6 months), ART status (ART-positive group included patients who had ART lasting for 3 months or more before the admission), opportunistic infections (OIs), serum HIV viral load (serum HIV viral load was classified as positive, negative or not done), history of syphilis treatment, serum toludine red unheated serum test (TRUST) titer (we analyzed the data using serum TRUST titer cutoff values of 1:16), serum *Treponema pallidum* particle agglutination (TPPA) qualitative result, CSF pressure, CSF TRUST result, CSF TPPA result, CSF white blood cell (WBC) count, CSF protein level, CSF glucose level, neurological symptoms and signs, imaging findings examined with cranial computerized tomography, and/or magnetic resonance imaging.

Those patients were divided into HIV/AIDS with neurosyphilis group (referred to as Neurosyphilis group) and HIV/AIDS with syphilis group (referred to as Syphilis group) according to the following diagnostic and exclusion criteria.

This study was approved by the Institution Ethics Committee of Beijing Youan hospital, Capital Medical University. The data were analyzed retrospectively and anonymously, the committee decided to waive the need for written informed consent from all participants.

### 2.2 Diagnostic criteria

The diagnosis of HIV/AIDS was based on Chinese guidelines for the diagnosis and treatment of HIV/AIDS (2018).^[12]^ The diagnostic criteria for neurosyphilis was based on Sexually Transmitted Diseases Treatment Guidelines (2015)^[13]^ and Treatment guidelines of Syphilis, Gonorrhea, Genital Herpes, and Genital Chlamydia Trachomatis Infection (2014).^[14]^ TRUST and TPPA tests were used instead of venereal disease research laboratory (VDRL) and FTA-ABS tests since these last 2 tests were not carried out in the 2 hospitals. The criteria for the diagnosis of neurosyphilis included positive serum TRUST test, positive serum TPPA test, and one or more of the following: positive CSF TRUST test; and positive CSF TPPA test, and CSF WBC count >20 × 10^6^/L. The criteria for the diagnosis of syphilis included positive serum TRUST test, positive serum TPPA test, negative CSF TRUST test, and one or more of the following: negative CSF TPPA test; and positive CSF TPPA test, and CSE WBC counts <20 × 10^6^/L.

Early syphilis included primary, secondary, and early latent syphilis. Late syphilis included late latent and syphilis of unknown duration. Patients with other concurrent CNS infections or caused by not syphilis were excluded from our study.

### 2.3 Statistical analysis

Continuous variables with normal distribution were assessed using the mean and standard deviation, or else using the median and inter quartile range. Categorical data were reported as number (n) (%). Continuous variables were analyzed using the Student *t* test. Categorical data were analyzed by the chi-square test. Multivariable analysis was performed with logistic regression. The overall multivariate logistic regression analysis was built by identifying, as potential predictors, all variables that either attained a significance level of less than 0.10 in the analyses. Next, a stepwise forward selection procedure was used to obtain a parsimonious model using a significance level for entry and exit from the model of 0.05. IBM SPSS 23.0 for Windows (IBM SPSS Inc., Chicago, IL) was used for all analyses. A 2-tailed *P* value <.05 was considered statistically significant.

## 3 Results

### 3.1 Demographic and clinical characteristics

A total of 81 HIV-infected patients were confirmed to have syphilis, divided 33 patients into the neurosyphilis group and 48 patients into the syphilis group. Most of the patients were middle-aged unmarried men. More than half of the patients were newly diagnosed with HIV/AIDS. Only 39 of patients (48.1%) had taken ART for more than 3 months before admission. Fifty-three of patients (65.4%) had OIs; 61 of patients (75.3%) had not been treated for syphilis. There were no statistically significant differences between the 2 groups in terms of age, gender, marital status, HIV course, ART, OIs, HIV viral load, and history of syphilis treatment (Table 1).

**Table 1:** Demographic and clinical characteristics of patients in neurosyphilis group and syphilis group.

Characteristics	Total (n = 81)	Neurosyphilis group (n = 33)	Syphilis group (n = 48)	*P* value
Age (mean ± SD, yr)	37.8 ± 11.8	39.8 ± 11.5	36. 5 ± 12.0	.206
Male (n, %)	78 (96.3)	32 (97.0)	46 (95.8)	1.000
Marital status (n, %)				.240
Unmarried	48 (59.3)	17 (51.5)	31 (64.6)	
Married	33 (40.7)	16 (48.5)	17 (35.4)	
HIV transmission route (n, %)				.010
Heterosexual contact	22 (27.2)	15 (45.5)	7 (14.6)	
Homosexual contact	41 (50.6)	11 (33.3)	30 (62.5)	
Intravenous use	1 (1.2)	0 (0)	1 (2.1)	
Not known	17 (21.0)	7 (21.2)	10 (20.8)	
HIV/AIDS course (n, %)				.694
Newly diagnosed	42 (51.9)	17 (51.5)	25 (52.1)	
Within 6 mo	14 (17.3)	7 (21.2)	7 (14.6)	
Longer than 6 mo	25 (30.9)	9 (27.3)	16 (33.3)	
ART status (n, %)	39 (48.1)	6 (18.2)	33 (68.8)	<.001
OIs (n, %)	53 (65.4)	19 (57.6)	34 (70.8)	.220
CD4 cell count M (IQR)	120 (31, 285)	90 (24, 251)	219 (48, 366)	.048
CD4 cell count (n, %)				.001
<200 cells /μL	54 (66.7)	29 (87.9)	25 (52.1)	
≥200 cells /μL	27 (33.3)	4 (12.1)	23 (47.9)	
Serum HIV viral load (n, %)				.102
≥1000 copies/mL	26 (72.7)	18 (90.0)	8 (66.7)	
<1000 copies/mL	6 (27.3)	2 (10.0)	4 (33.3)	
Previous untreated with syphilis (n, %)	61 (75.3)	31 (93.9)	30 (62.5)	.001
Serum TRUST titer (n, %)				.007
<1:16	31 (38.7)	7 (21.2)	24 (51.1)	
≥1:16	49 (61.3)	26 (78.8)	23 (48.9)	
Imaging findings (n, %)				.035
Abnormality	20 (32.8)	12 (48.0)	8 (22.2)	
Normal	41 (67.2)	13 (52.0)	28 (77.8)	
With symptoms and signs				
Yes	50 (61.7)	28 (84.8)	22 (45.8)	<.001
No	31 (38.3)	5 (15.2)	26 (54.2)	

However, the median CD4 cell counts of neurosyphilis group was 219 cells/μL, which was significantly higher than that of syphilis group (90 cells/μL), (*P* = .048). The proportions of CD4 cell count ≤100 cells/μL, 100 to 200 cells/μL, 200 to 500 cells/μL, ≥500 cells/μL between the 2 groups were significant difference (*P* = .048). There was also a significant difference in possible HIV transmission route between the 2 groups (*P* = .010). Heterosexual contact was the predominant transmission route in the neurosyphilis group (45.5%), while homosexual contact was the main transmission route in the syphilis group (62.5%).

### 3.2 Serum TRUST and TPPA test

Of the 80 patients had serum TRUST and TPPA test, 61.3 % (49/80) TRUST titers ≥1:16, 100% (80/80) with TPPA test positive. The proportion of serum TRUST titer ≥1:16 in the neurosyphilis group was significantly higher than that in the syphilis group (78.8% vs 48.9%), and the observed difference was statistically significant (*P* = .007) (Table 1).

### 3.3 Symptoms and signs

Fever was not a typical manifestation of syphilis or neurosyphilis, and it was usually caused by IOs among HIV/AIDS hospitalized patients. Therefore, fever and other neurological symptoms were calculated separately. The proportion of those with symptoms in the neurosyphilis group was significantly higher compared to those of the syphilis group (84.8% vs 45.8%; *P* < .001). The clinical manifestations of neurosyphilis sorted by the highest incidence included: the decline of muscle strength (7), paropsia (7), headache or dizziness (7), rash (6), abnormal ophthalmic examination (6), vomiting (4), heterophthongia (4), paresthesia (4), dysgnosia (4), hypoacusis (2), changes in consciousness (1), emotional lability (1), pathological signs (1), meningeal sign (1). Except lower percentage of incidence of fever (24.0% vs 48.0%; *P* = .030) and higher percentage of incidence of decline of muscle strength (21.2% vs 2.1%; *P* < .001) in the neurosyphilis group compared to the syphilis group, there was no significant difference in other manifestations between the 2 group (Table 2).

**Table 2:** Symptoms and signs of patients in neurosyphilis group and syphilis group.

	Neurosyphilis group (n = 33)	Syphilis group (n = 48)	*P* value
Symptoms			
Fever	8 (24.0)	23 (48.0)	.030
Decline of muscle strength	7 (21.2)	1 (2.1)	<.001
Paropsia	7 (21.2)	5 (10.4)	.18
Headache or dizziness	7 (21.2)	4 (8.3)	.10
Rash	6 (18.2)	6 (12.5)	.48
Abnormal ophthalmic examination	6 (18.2)	7 (14.6)	.66
Vomiting	4 (12.1)	0 (0.0)	.01
Heterophthongia	4 (12.1)	0 (0.0)	.01
Paresthesia	4 (12.1)	1 (2.1)	.07
Dysgnosia	4 (12.1)	3 (6.3)	.36
Hypoacusis	2 (6.1)	3 (6.3)	.97
Signs			
Conscious change	1 (3.0)	1 (2.1)	.79
Emotional lability	1 (3.0)	1 (2.1)	.79
Pathological signs	1 (3.0)	0 (0.0)	.22
Meningeal sign	1 (3.0)	0 (0.0)	.22
Seizures	0 (0.0)	1 (2.1)	.40

### 3.4 Imaging findings

Sixty-one of the patients took imaging examination. The findings were divided into abnormal and normal categories. There was no significant difference between the 2 groups (*P* = 3.350). Among the imaging findings of neurosyphilis patients, 39.4% were normal, 36.4% with abnormalities. The abnormalities manifested as low density infection foci (15.2%), ischemic infarction (15.2%), leukodystrophy (3.0%), and encephalatrophy (3.0%).

### 3.5 CSF examination

CSF WBC count (26.5 × 10^6^/L) and CSF protein level (786 mg/L) in the neurosyphilis group were significantly higher compared to those of the syphilis group (5.5 × 10^6^/L, 362 mg/L) (*P*< .001). There were no significant differences in CSF pressure, CSF glucose levels, CSF chloride, and CSF red blood cells (Table 3) between the 2 groups. Among 32 patients who completed CSF HIV viral load detection, HIV viral load in the neurosyphilis group (53,650 cells/μL) was significantly higher than that in the syphilis group (122 cells/μL) (*P* = .002).

**Table 3:** Cerebrospinal fluid abnormalities in neurosyphilis group and syphilis group.

	Neurosyphilis group (n = 33)	Syphilis group (n = 48)	*P* value
CSF pressure (mm Hg) (mean ± SD)	10.21 ± 4.46	9.34 ± 2.41	.318
CSF WBC count (×10^6^/L) M (IQR)	26.5 (6.8, 333)	5.5 (2, 49)	<.001
CSF protein level (mg/L) M (IQR)	786 (574, 3909)	362 (271, 994)	<.001
CSF glucose level (mmol/L) M (IQR)	2.9 (2.7, 3.4)	3.6 (2.8, 3.7)	.057
CSF chloride (mmol/L) M (IQR)	121.0 (118.5, 123.5)	121.7 (117.4, 124.1)	.769
CSF HIV viral load (cells/μL) M (IQR)	53,650 (7290, 155,500)	122 (0, 1772.5)	.002

### 3.6 Risk factors associated with the occurrence of neurosyphilis

In univariate analysis, we found possible HIV transmission route, ART status, CD4 cell count, serum TRUST titer, previous untreated with syphilis, and were related to the occurrence of neurosyphilis.

In multivariate logistic regression analysis, the risk factors associated with occurrence of neurosyphilis were heterosexual contact as possible HIV transmission route (odds ratio [OR] 8.171; 95% confidence interval [CI]: 1.363–48.974), CD4 cell count <200 cells/μL (OR 1.486; 95% CI: 1.012–6.651), previous untreated with syphilis (OR 2.853; 95% CI: 1.114–6.249), and serum TRUST titer ≥1:16 (OR 5.580; 95% CI: 1.492–20.235). The protective factor associated with occurrence of neurosyphilis was received ART (OR 0.451; 95% CI: 0.037–1.069) (Table 4).

**Table 4:** Risk factors associated for the occurrence of neurosyphilis among HIV infected patients.

Risk factors	Neurosyphilis group (n = 33)	Syphilis group (n = 48)	OR	95% CI	*P* value
Transmission route					
Homosexual contact	11	30	1.000		
Heterosexual contact	15	7	8.171	1.363 to 48.974	.022
ART status					
No	27	15	1.000		
Yes	6	33	0.451	0.037 to 1.069	.013
CD4 cell count					
<200	29	25	1.486	1.012 to 6.651	.037
≥200	4	13	1.000		
Previous untreated with syphilis					
Yes	2	18	1.000		
No	31	30	2.853	1.114 to 6.294	.015
Serum TRUST titer					
<1:16	7	24	1.000		
≥1:16	26	23	2.381	1.027 to 8.247	.001
With neurological symptoms and signs					
No	5	26	1.000		
Yes	28	22	5.580	1.492 to 20.235	.010

## 4 Discussion

Syphilis and HIV/AIDS had the same route of transmission and affect the same group of high-risk populations. It reported that HIV/AIDS patients were 77 times more likely to be infected with syphilis than HIV-negative ones.^[15]^ The co-infection rate among HIV/AIDS patients in China was about 9%.^[16]^ HIV infection severely damaged the immune system, which in turn accelerates the natural course of syphilis, resulting in the shortened incubation period, atypical symptoms, and increased incidence of neurosyphilis.^[17]^ Both humoral immunity and cellular immunity of HIV/AIDS patients are inhibited to varying degrees, and HIV can cause meningeal lesions, making it easier for *TP* to cross the blood–brain barrier. Therefore, HIV/AIDS patients are more prone to neurosyphilis when they are co-infected with *TP*. Farhi and Dupin^[18]^ reported that the incidence of neurosyphilis in HIV-infected population was about 0.6%∼16%. A study by Lynn and Lightman^[4]^ found that the incidence of neurosyphilis in untreated HIV-positive syphilis patients without ART was 23.5%, while the incidence of neurosyphilis in untreated HIV-negative syphilis patients was approximately 10%. At the same time, the majority would lack symptoms and spontaneously clear the infection from the CSF without requiring treatment. However, this spontaneous remission may be more difficult to occur in HIV/AIDS patients due to immune deficiency, leading to a high incidence of neurosyphilis.

In order to further understand the clinical characteristics of HIV/AIDS and neurosyphilis co-infection, we comparatively analyzed clinical data of HIV/AIDS patients with neurosyphilis and syphilis. The proportion of serum TRUST titer ≥1:16 was significantly higher in the neurosyphilis group, while the *P*-value in the multivariate logistic regression analysis was significant. Several studies suggested that the serum RPR titer >1:32 was closely related to the occurrence of neurosyphilis,^[19,20]^ while others used 1:128 as a threshold.^[7]^ In our study, the serum TRUST titer, as one of the indicators, of the neurosyphilis group was significantly higher than that of the syphilis group.

The levels of CSF WBC (26.5 vs 5.5 × 10^6^/L), CSF protein (786 vs 362 mg/L), and CSF HIV viral load (53,650 vs 122 cells/μL) of the neurosyphilis group were significantly higher than those of the syphilis group. *TP* could invade various organs of the body, including the CNS, cause transient or persistent inflammation, which manifested as abnormal CSF examination. CSF WBC count was an indicator used to diagnose neurosyphilis when it exceeded 20 × 10^6^/L in HIV/AIDS patients. It was very sensitive but with low specificity. CSF proteins were usually elevated in patients with neurosyphilis; however, they neither had sensitivity nor specificity.^[21]^ The previous study showed that the CSF WBC count and protein were higher in symptomatic neurosyphilis patients compared to asymptomatic ones, while most of the symptomatic patients had abnormal results of CSF examination.^[22]^ Another study showed that CSF protein was closely related to the presence of symptoms.^[23]^ Syphilis infection can temporarily increase HIV replication, and neurosyphilis may amplify HIV viral load in CSF, which may be a consequence of intrathecal immune activation and subsequent amplification of HIV replication.^[24]^ Our results showed that the CSF WBC, CSF protein, and CSF HIV viral load in the neurosyphilis group were significantly higher compared to the syphilis group, which was consistent with the results from previous studies.^[25]^ Since most of the patients in our study were not treated with ART, there was a more remarkable change of these indicators between the 2 groups. There were no significant differences in CSF pressure, CSF glucose level, and CSF chloride between the 2 groups. Since no previous studies reported on these indicators, we assumed they did not significantly change in neurosyphilis patients.

Our study showed that HIV transmission route between the 2 groups was significantly different. The main transmission route in the neurosyphilis group was heterosexual contact, while in the syphilis group, it was homosexual contact. Heterosexual contact was a risk factor related to the occurrence of neurosyphilis. Heterosexual contact was identified as the main transmission route for HIV/AIDS in China since 2007,^[26]^ while HIV/AIDS caused by homosexual contact has been increasing over recent years.^[27]^ Out of the 81 patients included in the present study, 96.3% were male, and 50.6% were infected through homosexual contact, which was in line with previous reports.^[11,28]^ In our study, most of the patients from the neurosyphilis group were infected through heterosexual contact, which was related to the occurrence of neurosyphilis. This might be explained by the fact that the gay population was generally younger, with a higher education level, and thus was likely to do more regular screening of HIV and *TP* regardless of the absence of symptoms, and treat syphilis earlier. The patients with heterosexual contact usually were diagnosed and treated at the late period of not receiving test timely. It could also be due to the small sample size, which should be addressed by further research.

Another high-risk factor for neurosyphilis was the presence of neurological symptoms and signs. The 2014 European Syphilis Management Guidelines regarded neurological symptoms as an indication for performing a lumbar puncture since the incidence of neurosyphilis rose in a situation, which was consistent with our result. In the neurosyphilis group, 84.8% of patients had neurological symptoms and signs. The decline of muscle strength, paropsia, headache or dizziness, rash, abnormal ophthalmic examination, vomiting, heterophthongia, paresthesia, and dysgnosia all occurred at an incidence that exceeded 10%. These data emphasized the importance of detailed consultation and physical examination in clinical work, as well as further examination and treatment that should be timely performed when these positive manifestations were observed. Detailed consultation and physical examination resulted as the most cost-effective diagnostic methods, which should be valued by clinicians.

Syphilis infection can decrease CD4 cell counts and increase virus replication in HIV/AIDS patients. CD4 cell counts less than 200 cells/μL, serum RPR/VDRL titers ≥1:32 suggested a significant increase in the incidence of neurosyphilis, which needed to be addressed by further lumbar puncture examination.^[19,20]^ In the present study, both the CD4 cell count and the percentage of CD4 cell count more than 200 cells/μL that were found in the neurosyphilis group were lower compared to the syphilis group. This might be because the proportion of received ART in the neurosyphilis group was higher than that of the syphilis group, and standardized ART can help increase the level of CD4 cell count and not received ART increased the risk of neurosyphilis.^[29]^ This result was similar to the study Salado et al^[7]^ which indicated that patients with syphilis with a CD4 cell count <350 cells/μL, male, and not receiving ART were risk factors for developing neurosyphilis in patients with HIV infection.

The imaging findings of neurosyphilis tended to lack specificity; they varied from normal to a variety of manifestations such as abnormalities in the cerebral cortex and white matter of the brain, atrophy of the brain, meningeal enhancement, and cerebrovascular lesion. A previous study analyzed the imaging findings from 35 neurosyphilis patients, of which 32 were HIV-positive; 11 (31%) had normal imaging findings, 8 (23%) had cerebral infarction, 7 (20%) had nonspecific white matter lesions, 2 (6%) showed meningitis and off-axis enhancement suggesting meningitis.^[30]^ Our results were similar to that; there were 39.4% neurosyphilis patients with normal imaging findings, and 36.4% with abnormalities, including low-density infection (15.2%), ischemic infarction (15.2%), white matter lesions (3.0%), and brain atrophy (3.0%).

It should be noted that the diagnosis of neurosyphilis depended on clinical manifestations, syphilis serology, and CSF examination. The diagnostic criteria slightly varied in different guidelines. The CSF VDRL experiment had high specificity but lacked sensitivity, while the CSF FTA-ABS had high sensitivity but poor specificity.^[3]^ Since these 2 tests were not performed at the hospitals of the study, the CSF TRUST and CSF TPPA experiments were used as a replacement for CSF VDRL and CSF FTA-ABS. Because HIV virus could cross the serum–brain barrier, and elevate the CSF WBC and CSF protein level, the CSF WBC count> 20 × 10^6^/L was selected as the abnormal standard according to the 2015 US guidelines.^[31]^ China's 2014 guidelines defined CSF protein >500 mg/L as a threshold for abnormality,^[13]^ while the normal range of CSF protein at our hospital was 0 to 600 mg/L. We could not define CSF protein >500 mg/L as a criterion to diagnose neurosyphilis. Since we did not find any other criteria for the diagnosis of neurosyphilis with CSF protein, to ensure the diagnostic accuracy of neurosyphilis, CSF protein was not included in the diagnostic criteria in the present study.

There were some limitations in this study. First, since our study was a retrospective one, it was difficult to control the baseline of included patients. Some data such as HIV possible transmission route were not acquired in many patients, and some tests such as serum HIV viral load, CSF HIV viral load, and imaging examination were not performed for a high number of patients. The sample size of our study was small, only in-patients, and thus could lead to insufficient test efficiency in statistical analysis, which should be addressed by further multi-center studies. Second, the diagnostic criteria in this study was set on serum TRUST and TPPA test values with 1 or more other results of positive CSF test, and CSF TRUST and CSF TPPA tests were used as a replacement for CSF VDRL and CSF FTA-ABS. Other diagnosis guidelines favored VDRL and TPPA findings for the diagnosis of neurosyphilis. Therefore, generalizability is limited where these lab tests are performed.

## 5 Conclusion

Lower CD4 cell count, higher serum TRUST titer, HIV transmitted by heterosexual contact, not received ART, and untreated with syphilis, were associated with neurosyphilis among HIV/AIDS patients with syphilis. The presence of neurological symptoms and signs prompted the possibility of neurosyphilis occurrence. Lumbar puncture and CSF testing for the diagnosis of neurosyphilis is required in patients infected with HIV and syphilis.

## Author contributions

LA, YJH conceived and coordinated the study, designed, performed, and analyzed the experiments, wrote the draft. SJC, WH, LJW, SY, YJZ, DLL, and WXW carried out the data collection, data analysis, and revised the manuscript. All authors reviewed the results and approved the final version of the manuscript.

**Conceptualization:** An Liu.

**Data curation:** Jianhua Yu, JinChuan Shi, Hu Wan, Jianwei Li, Ying Shao, Jiangzhu Ye, Lili Dai, An Liu.

**Formal analysis:** Jianhua Yu, JinChuan Shi, Hu Wan, Jianwei Li, Jiangzhu Ye, Lili Dai.

**Investigation:** JinChuan Shi, Hu Wan, Jianwei Li, Ying Shao, Jiangzhu Ye, Lili Dai, An Liu.

**Methodology:** Hu Wan, Jianwei Li, Ying Shao, Jiangzhu Ye, Lili Dai, Xiwen Wang, An Liu.

**Project administration:** Lili Dai, Xiwen Wang, An Liu.

**Software:** Jianhua Yu, JinChuan Shi, Xiwen Wang.

**Validation:** Jiangzhu Ye, Xiwen Wang.

**Writing – original draft:** Jianhua Yu.

**Writing – review & editing:** An Liu.
